# A case report of soluble A antigen confounding the blood type in a haplo‐identical hematopoietic stem cell transplant patient with graft‐versus‐host disease

**DOI:** 10.1111/trf.70221

**Published:** 2026-04-10

**Authors:** Sheri Hugan, Nada Naiyer, Laura Cooling

**Affiliations:** ^1^ Michigan Medicine University of Michigan Ann Arbor Michigan USA; ^2^ Corewell Health Grand Rapids Michigan USA

**Keywords:** ABO, allogeneic transplant, GVHD

## Abstract

**Background:**

ABO‐incompatible (ABOi) hematopoietic stem cell transplantation (HSCT) is often associated with ABO discrepancies post‐HSCT. Re‐emergence of the patient's original type post‐HSCT may signal graft loss, disease relapse, recent transfusion, or soluble ABO antigen. We present a group A pediatric patient with leukemia who underwent a minor ABOi HSCT from a group O donor with persistent and increasing A reactivity 2 years post‐HSCT.

**Methods:**

The patient's electronic medical and laboratory records were reviewed. ABO, Lewis typing, and direct antiglobulin (DAT) tests were performed by column agglutination and/or tube method. Soluble anti‐A was demonstrated by adsorption and human anti‐A neutralization. Elution of soluble A antigen was performed by incubation of patient red cells with group A_1_, Le(a + b‐) plasma.

**Results:**

The patient typed as Le(a‐b+) and demonstrated 1+ reactivity with anti‐A reagent, which increased (4+) during an episode of gastrointestinal graft‐versus‐host disease (GVHD). There was no evidence of disease relapse with 100% donor cells by molecular testing, normal blood counts, and a negative DAT. A high level of soluble A‐antigen in patient plasma was demonstrated by adsorption and anti‐A neutralization studies. Finally, soluble A antigen was successfully eluted from patient RBCs to reveal only group O donor type RBCs.

**Discussion:**

We present a case of persistent A antigen post‐ABOi HSCT due to soluble A antigen of patient origin. Soluble A antigen was demonstrated using adsorption, neutralization studies, and a modified elution method previously described for Lewis antigens. This case suggests increased soluble ABO antigen in the setting of gut GVHD.

**Abbreviations**
ABOi: ABO incompatible
CAT: column agglutination
ECP: extracorporeal photopheresis
GVHD: graft versus host disease
HSCT: hematopoietic stem cell transplantation

## 1 INTRODUCTION

Unlike organ transplantation, ABO is not a barrier to allogeneic hematopoietic stem cell transplantation (HSCT) but can present challenges in the post‐HSCT setting for testing and transfusion support.^1^ Major ABO incompatibility (ABOi) can be associated with hemolysis, delayed erythroid engraftment, and pure red cell aplasia.^1^ Hemolysis can also be observed with minor ABOi HSCT due to high titer isoagglutinins in donor plasma and donor lymphocyte syndrome.^1^ ABOi is frequently associated with ABO discrepancies and may increase the risk for graft‐versus‐host‐disease (GVHD).^2^, ^3^ Post‐HSCT, the re‐emergence of patient's native ABO type post‐HSCT can be an early indicator of relapse or graft loss.^4^ We present a case study of a minor ABOi HSCT patient with a strong reemergence of the patient's ABO type attributed to high levels of soluble ABO antigen in the setting of severe GVHD. It is well known that transfused red cells will become the same Lewis type as the recipient over time. In 1955, Sneath and Sneath conducted experiments to document the conversion of Lewis antigens. They tested one volume of packed red cells shaken continuously in 10 volumes of plasma at 35°C for at least 3 days, with the plasma being changed daily.^5^ We applied this method to remove soluble ABO antigen to help distinguish between re‐emergence of recipient erythropoiesis versus passive adsorption of soluble ABO antigen on engrafted donor red cells.

## 2 CASE PRESENTATION

**FIGURE 1:**
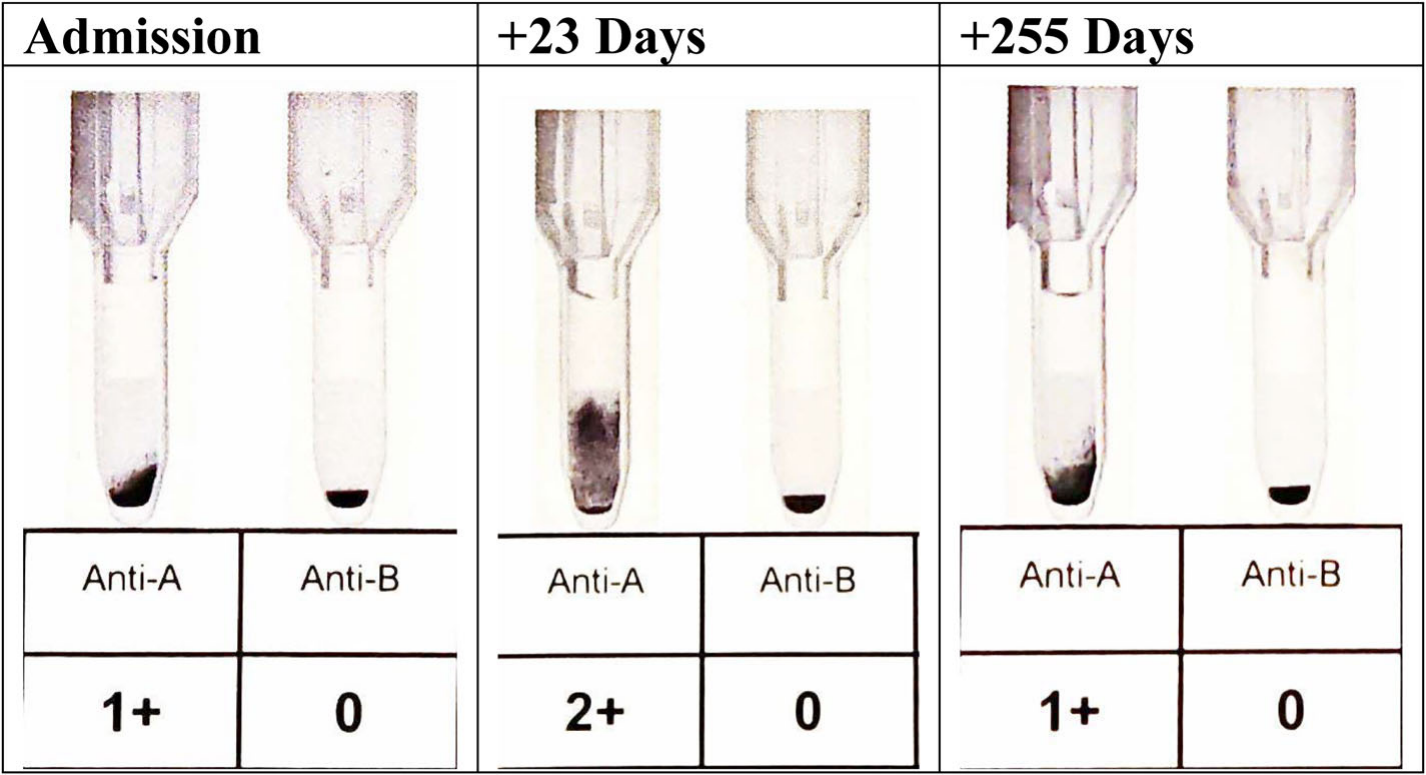
Blood type on column agglutination test at admission, peak GVHD, and recovery. Group A, Rh(D) positive B‐ALL patient and Group O, Rh(D) positive donor blood type column agglutination results for anti‐A and anti‐B in the patient 2 years post‐HSCT. Results show weak anti‐A reactivity (1+) at admission for GVHD, which increased to 2+ as GVHD worsened. After treatment for GVHD, the A‐reactivity returned to baseline weak positive.

The patient was an 11‐year‐old, group A, Rh(D) positive male with a history of relapsed pre‐ B‐cell acute lymphoblastic leukemia, who underwent a minor ABOi, haploidentical HSCT from a group O, Rh(D) related donor. His early post‐HSCT course was unremarkable with early platelet and myeloid engraftment by days 13 and 15, respectively. Molecular studies confirmed full donor cell engraftment with 100% CD3+ lymphoid cells and 100% CD33+/CD66b + myeloid cells at day 32. His late post‐transplant course was complicated by grade II chronic GVHD involving the gastrointestinal tract (GI biopsy confirmed, stage 1) and lung (CT chest confirmed). The patient was admitted and treated with 2 mg/kg methylprednisolone, 1 mg tacrolimus, 5 mg ruxolitinib twice daily, and 15 cycles of extracorporeal photopheresis (ECP). He was transfusion‐independent but did require 1 unit of RBC on 3 occasions to prime the ECP circuit. Despite full engraftment, the patient's forward typing showed persistent trace group A RBCs post‐HSCT, which increased markedly during admission for GVHD (Table 1; Figure 1). Mixed field reactions may be seen due to varying concentrations of soluble antigen in plasma adhering to group O red cells. Molecular studies confirmed full donor cell engraftment at 1‐ and 2‐year post‐HSCT, with no evidence of disease relapse by flow cytometry and bone marrow biopsy.

**TABLE 1:** Patient ABO typing during the case study.

Days from admission^a^	Method		Forward			Reverse	
	CAT^b^	Tube^c^	Anti‐A	Anti‐B	Anti‐A,B	A_1_ cells	B cells
+3 days	X		1+	0		0	2+
+7 days	X		±	0		0	2+
+14 days	X		1+	0		0	3+
+23 days	X		2+	0		0	2+
+23 days		X	4+ Mixed field	0	3+ Mixed field	0	2+
+30 days	X		2+	0		0	2+
+255 days	X		1+	0		0	2+

^a^ Admission for gastrointestinal GVHD, over 2 years after allo‐HSCT.

^b^ Reagents used with Column Agglutination Testing from QuidelOrtho using anti‐A (clone BIRMA‐1), anti‐B (clone LB‐2), and 0.8% pooled reverse group cells.

^c^ Reagents used with tube method from Werfen anti‐A (clone BIRMA‐1), anti‐B (clone GAMA110), and anti‐A,B (clones Birma‐1, ES4, and ES15), and QuidelOrtho 2%–4% pooled reverse group cells.

## 3 METHODS

### 3.1 Serologic tests and data collection

Routine ABO and RhD typing were performed via automated column agglutination technology (CAT) on the Ortho Vision Max using MTS A/B/D monoclonal and reverse grouping cards (QuidelOrtho, Raritan, NJ) and tube method used reagents from Werfen, Norcross, GA. Murine monoclonal (IgM) anti‐A (BIRMA‐1), anti‐B (LB‐2), and anti‐D (MS‐201) were used in CAT. Murine monoclonal (IgM) anti‐A (BIRMA‐1), anti‐B (GAMA110), and anti‐D (MS‐201) were used in the tube method. Monoclonal anti‐A,B (Birma‐1, ES4, and ES15), blood grouping control, and 2%–4% pooled reagent group O and A_1_ red cells were purchased from Werfen. Lewis phenotyping was performed by tube method using monoclonal anti‐Le^a^ and anti‐Le^b^ from Werfen. Dolichos biflorus lectin, rabbit anti‐human globin (AHG), Coombs control cells, and 2%–4% reverse group A_1_ and B cells were purchased from QuidelOrtho. Reagent human plasma from group A_1_, Le(a + b‐); group A_1_, Le(a‐b+); and group B with a high‐titer anti‐A were obtained by screening routine patient samples from the blood bank. A review of the patient medical records was performed.

### 3.2 Elution of soluble A antigen

Soluble group A antigen was eluted from patient red cells by mass action using a procedure previously described for Lewis antigens.^5^, ^6^ Patient red cells (100 μL packed cells) were incubated 1:10 vol/vol with 1 mL of plasma from a group A_1_, non‐secretor (Le(a + b‐)). Group A_1_ plasma was selected to lack soluble A antigen and be compatible with both the HSCT donor and recipient. A standard blood bank water bath set to 37°C was used instead of 35°C as stated in the original experiments. Elution occurred over a period of 6 days which was within the guidelines of the original paper of more than 3 days. Cells were then washed, resuspended, and forward blood type tested using monoclonal typing reagents (Werfen) by tube method. Blood type controls did not detect any measurable changes in the red cell membrane after elution.

### 3.3 Adsorption of soluble A antigen

To demonstrate the presence of group A antigen in the patient plasma, 100 μL packed group O reagent red cells were incubated with 1 mL patient plasma (1:10 vol/vol) in a 37°C water bath for 4 days with daily plasma replacement.^5^, ^6^ As a control, group O reagent red cells were also incubated with 1 mL plasma from an A_1_, Le(a + b‐) non‐secretor. On day 5, cells were washed, resuspended, and forward blood typing was tested as before.

### 3.4 Neutralization of soluble A antigen (AHG)

Patient plasma (1 mL) was incubated with human anti‐A (1 mL) from a high‐titer (1:512), group B individual in a 37°C water bath for 30 min. Controls included (1) patient plasma incubated with saline and (2) high‐titer, human anti‐A incubated with plasma from a random group A secretor (Le(a‐b+)). Following incubation and neutralization, plasma samples underwent a series of doubling dilutions in saline for titration against commercial A_1_ reagent red cells. Anti‐A IgM titers were defined as a 1+ agglutination after 15‐min room temperature incubation.^7^ Anti‐A IgG titers were determined after 15‐min 37°C incubation, washing (×4) and AHG.

## 4 RESULTS

The persistence and increase in A antigen on patient red cells, despite full donor engraftment and no evidence of disease relapse, suggested passive adsorption of soluble A antigen. Soluble ABO antigen found in plasma and secretions is the product of the non‐erythroid, *FUT2/Secretor* gene and may persist after engraftment in ABOi HSCT.^6^, ^8^, ^9^, ^10^, ^11^, ^12^, ^13^, ^14^, ^15^ Serologic Lewis typing confirmed that the patient was *FUT2*+ (Leb+) and capable of synthesizing soluble A antigen.^6^

A literature review did not yield a serologic method to distinguish group A red cells from group O red cells with adsorbed A antigen; however, a method for removal of soluble Lewis antigens is described.^5^, ^6^ To elute soluble A antigen, patient packed red cells were incubated 1:10 vol/vol with plasma from an A_1_, Le(a + b‐) non‐secretor specimen for 6 days with daily replacement of fresh reagent plasma. Soluble A antigen was successfully removed with no detectable A antigen on patient red cells (Table 2).

**TABLE 2:** Testing results.

Test	Red cells^a^	Antisera^b^	Pre	Post
			Agglutination	
Mass action elution of soluble A antigen with human A_1_, Le (a + b‐) plasma	Patient	Anti‐A	4+	0
		Anti‐B Control	0	0
		Anti‐D Control	4+	4+
			Agglutination	
Adsorption soluble A antigen using patient plasma	Group O	Anti‐A Anti‐A,B	0 0	1+ 2+
Adsorption soluble A antigen using human A_1_, Le(a + b‐) Plasma (Control)	Group O	Anti‐A Anti‐A,B	0 0	0 0
			Endpoint	
IgM titration endpoint results to demonstrate neutralization of high‐titer, human Anti‐A	Group A_1_	Patient Plasma	1: 512	1: 32
		A_1_, Le (a‐b+) Plasma Control	1: 512	1: 128
Saline control	Group A_1_	Patient Plasma	1: 512	1: 512
			Endpoint	
IgG titration endpoint results to demonstrate neutralization of a high‐titer, human Anti‐A	Group A_1_	Patient Plasma	1: 512	1: 64
		A_1_, Le (a‐b+) Plasma Control	1: 512	1: 256
Saline control	Group A_1_	Patient Plasma	1: 512	1: 512

^a^ Reagent red cells used with tube method from QuidelOrtho: Group O and A1.

^b^ Reagents used with tube method from Werfen: anti‐A (clone BIRMA‐1), anti‐B (clone GAMA110), anti‐D (clones MS201 and MS26), and anti‐A,B (clones Birma‐1, ES4, and ES15).

To confirm the presence of soluble A antigen in patient plasma, reagent group O cells were then incubated with patient plasma.^6^, ^11^, ^14^ As a control, group O cells were incubated in parallel with reagent plasma from the previously described A_1_, non‐secretor individual. To detect adsorbed A antigen, cells were washed and tested with reagent anti‐A. As shown, group O cells showed A reactivity after incubation with patient plasma but not with the non‐secretor plasma control (Table 2).

Finally, we performed a neutralization of the patient's plasma using human anti‐A from a high‐titer (1:512), group B individual. To assess neutralization, anti‐A was titrated and compared to a saline control. As an additional control, human anti‐A was also incubated with plasma from a group A_1_, Leb + individual without GVHD. As shown in Table 2, anti‐A titer was significantly lower after incubation with patient plasma than either the saline or a group A secretor controls. The lower titer observed with patient plasma supports a higher than normal level of soluble A antigen.

## 5 DISCUSSION

Serologic and hematologic issues following ABOi HSCT are well recognized including ABO discrepancies, hemolysis, delayed erythroid engraftment and pure red cell aplasia.^1^, ^2^ Transfusion support post‐HSCT requires provision of components compatible with both donor and recipient ABO types, which can complicate laboratory evaluation for erythroid engraftment.^1^, ^2^ As a result, HSCT patients are typically evaluated for a blood type change to the donor type after 1 year post‐HSCT.^1^ Institutional criteria for changing ABO type include (1) absence of disease relapse, (2) full engraftment by bone marrow biopsy and molecular chimerism, (3) RBC transfusion independence, and (4) no mixed field or unexpected reactivity in routine blood typing. Additional laboratory findings supporting successful engraftment include a negative DAT and normal complete blood count.^1^

We present a case of strengthening recipient ABO type, despite full donor engraftment, due to high levels of soluble A antigen with adsorption onto donor group O red cells. The persistence of A antigen expression prohibited any change in ABO type and raised concerns for graft stability and/or disease relapse. Soluble A antigen in plasma was demonstrated by (1) Lewis phenotyping, (2) passive adsorption onto group O reagent red cells, and (3) neutralization of human anti‐A with patient plasma. Finally, to confirm that A expression on patient red cells was due to soluble A antigen, we modified a method described for removing Lewis antigen from cells.^5^, ^6^ Specifically, patient red cells were sequentially incubated with plasma from a group A_1_, Le(a + b‐) nonsecretor to displace and elute A‐active glycolipid from the membrane. After 6 days, the patient's red cells typed as group O donor type (Table 2). To our knowledge, this technique has never been described for ABO discrepancies post‐HSCT due to soluble ABO antigen.

Soluble ABO antigens, like Lewis antigens, are type 1 chain glycosphingolipid (GSL) antigens synthesized by intestinal epithelial cells and the *FUT2/Secretor* gene.^6^ It is estimated that 5% of ABO antigen on red cells is adsorbed from plasma.^16^ Soluble ABO antigens are responsible for weak ABO expression in the *para*‐Bombay type.^6^ Likewise, in ABOi HSCT, recipient‐type soluble ABO GSL in plasma can be absorbed onto donor‐derived RBC leading to weak ABO reactivity and clinical concerns regarding engraftment.^17^

Several prior case reports have also described “persistent” recipient ABO type in ABOi HSCT patients, particularly in pediatric patients.^8^, ^9^, ^10^, ^11^, ^12^, ^13^, ^14^, ^15^, ^17^ A_1_, Le(a‐b+) patients, but not A_2_ individuals, represent most cases in the current literature.^8^, ^11^, ^15^ In two small case series, up to 30% of recipients can show trace/weak residual ABO antigen on red cells using flow cytometry and absorption/elution of anti‐A.^11^, ^13^ Unlike re‐emergence of recipient erythropoiesis, which shows a dual RBC population, absorbed soluble A antigen is characterized by a single population of cells with weak A expression by flow cytometry.^10^, ^11^, ^14^, ^15^ Of note, the detection of weak soluble ABO antigen is reagent dependent and may require using anti‐A,B.^9^, ^12^, ^13^, ^15^

One unexpected finding in our case was the increase in A‐reactivity (2+) during an episode of intestinal GVHD, which returned to weak (1+) reactivity as GVHD subsided. These novel observations suggest *FUT2* upregulation with GVHD. A potential role for *FUT2* is reported in Crohn's disease and necrotizing enterocolitis, two inflammatory bowel diseases, and bacterial translocation and infection post‐HSCT.^18^, ^19^, ^20^, ^21^ In mouse models, *FUT2* can be upregulated by interleukin 22 and butyrate.^21^, ^22^ Another consideration for an increase in A‐reactivity may be due to intestinal epithelial cell damage by GVHD that may lead to endogenous lipoproteins to be released into the plasma. Moreover, early clinical trials of butyrate and interleukin 22 in allogeneic HSCT patients show promise in the prevention and treatment of gut GVHD, respectively.^23^, ^24^, ^25^

## 6 CONCLUSION

In an ABOi HSCT patient with severe gut GVHD, the rapid increase in recipient‐type A antigen raised concern for early signs of disease relapse.^4^ Prior case reports have described low level recipient ABO expression on engrafted donor RBCs due to soluble A antigen.^8^, ^9^, ^10^, ^11^, ^12^, ^13^, ^14^, ^15^ To differentiate adsorbed soluble ABO antigen from intrinsic red cell antigen expression, we adopted a previously described technique for Lewis antigen removal, with elimination of A reactivity.^5^, ^6^ The severity of gut GVHD appeared to stimulate increased soluble A antigen in this patient.

## FUNDING INFORMATION

The authors received no funding for this study.

## CONFLICT OF INTEREST STATEMENT

None of the authors have a conflict of interest to disclose.

## Data Availability

The data that support the findings of this study are available from the corresponding author upon reasonable request.
